# Large language model-generated clinical summaries in emergency departments: A blinded comparison study

**DOI:** 10.1371/journal.pdig.0001491

**Published:** 2026-07-09

**Authors:** Niloufar Golchini, Nikita Mehandru, Ahmed Alaa, Melanie Molina

**Affiliations:** 1 Department of Computational Precision Health, University of California Berkeley and San Francisco, Berkeley and San Francisco, California, United States of America; 2 Bakar Computational Health Sciences Institute, University of California San Francisco, San Francisco, California, United States of America; 3 Department of Emergency Medicine, University of California San Francisco, San Francisco, California, United States of America; 4 Department of Medicine, Division of Clinical Informatics and Digital Transformation, University of California San Francisco, San Francisco, California, United States of America; Australian Institute of Health Innovation, AUSTRALIA

## Abstract

Emergency department (ED) clinicians routinely construct “one-liner” summaries, distilling a patient’s history and presentation into one high-yield sentence that supports rapid decision-making. Producing these summaries is cognitively demanding. Large language models (LLMs) may assist by synthesizing longitudinal electronic health record (EHR) data. In this blinded study of 99 ED encounters, emergency physicians evaluated paired LLM- and physician-authored summaries on accuracy, completeness, and clinical utility, indicating their overall preference with free-text explanation. LLM-generated one-liner summaries were produced using k-nearest-neighbor few-shot prompting. We used linear mixed-effects to compare ratings. We also examined the LLM’s selective note inclusion and used rapid content analysis to summarize free-text explanations. We found that across all dimensions, LLM-generated summaries received higher ratings than physician-authored summaries. Mean (SE) estimated marginal means for accuracy were 4.18 (0.09) vs 3.40 (0.11) (β = 0.78; 95% CI 0.50–1.07), for completeness 3.69 (0.10) vs 3.25 (0.12) (β = 0.44; 95% CI 0.14–0.74), and for clinical utility 3.88 (0.10) vs 3.21 (0.12) (β = 0.67; 95% CI 0.35–0.99). The LLM utilized 53.2% of available notes, with History & Physical (H&P) notes requested in 97.0% of cases and imaging reports in 81.8%, demonstrating context-sensitive document selection that varied significantly by chief complaint. Qualitative analysis indicated that LLM summaries were often more inclusive and neutrally phrased, whereas physician summaries exhibited greater contextual nuance but occasionally omitted key details. These findings represent an important first step in evaluating whether LLMs can produce clinically acceptable one-liner summaries from longitudinal EHR data; prospective validation is required before deployment in real-world ED workflows.

## Author summary

Emergency physicians face the challenge of rapidly synthesizing a patient’s entire medical history — often spanning years of records across multiple hospitalizations — within minutes of meeting them for the first time. To support this process, physicians write a ‘one-liner’: a single sentence that distills the most relevant aspects of a patient’s background and current complaint. Crafting this summary is cognitively demanding, particularly in busy emergency departments where interruptions are frequent and time is scarce. In this study, we evaluated whether a large language model (LLM) — a type of artificial intelligence trained on vast amounts of text — could generate these one-liner summaries from electronic health record data. We asked 21 emergency physicians to blindly rate paired LLM- and physician-authored summaries across accuracy, completeness, and clinical utility. LLM-generated summaries received higher ratings across all three dimensions. Importantly, the two approaches showed complementary strengths: LLM summaries were more faithful to the medical record, while physician summaries contributed contextual nuance. These findings suggest that a hybrid approach, where LLMs provide a structured foundation that clinicians then refine, may represent the most promising path forward. Prospective validation in live clinical settings is needed before these tools can be deployed.

## Introduction

Emergency physicians make rapid, high-stakes decisions amid persistent time pressures, resource constraints, frequent interruptions, and emergency department crowding [1,2]. Upon first meeting a patient, they must quickly integrate clinical data from multiple sources within a patient’s electronic heath record (EHR) into a coherent mental model that supports diagnostic reasoning and immediate management. A key early step in this process is constructing a concise “one-liner”—an ED-specific clinical summary that distills the most important aspects of a patient’s demographics, medical history, and chief complaint into a single interpretive statement that typically opens the “History of Present Illness (HPI)” section of the clinical note. Unlike clinical settings where physicians know their patients longitudinally, ED physicians encounter patients without prior familiarity, making the rapid synthesis and summarization of relevant clinical information especially critical for situational awareness. In addition to facilitating formal handoff and documentation, the ED one-liner functions as a cognitive scaffold: it concisely summarizes relevant clinical context and frames the patient’s presenting complaint [3,4]. Because the one-liner often represents the ED physician’s first cognitive synthesis of the patient’s clinical history and presentation, its quality and accuracy can shape initial diagnostic reasoning and influence subsequent decisions [5].

Crafting a high-quality one-liner is cognitively demanding. On average, ED physicians are interrupted 12–13 times per hour, which makes navigating complex longitudinal records authored by multiple providers across varied care settings a challenging task [6]. These records often contain diverse note types (e.g., discharge summaries, consult notes, imaging reports) from different institutions, requiring ED physicians to search and filter through specific pieces of information [7]. Such cognitive labor competes directly with bedside patient care and contributes to fatigue and burnout [8]. Yet the cost of omission or misrepresentation in an ED one-liner can be clinically consequential, particularly when decisions must be made under time pressure and with limited information [9].

Large language models (LLMs) have recently shown strong performance in clinical reasoning and summarization tasks across a range of benchmarks and use cases [10–12]. Clinical applications have focused on generating long-form summaries, such as hospital discharge summaries, using notes from single encounters as inputs [13–16]. However, no study to date has systematically evaluated the ability of LLMs to generate concise “one-liner” summaries, which would require integrating multiple EHR information types (e.g., clinical notes, imaging reports) and clinical encounters across time. This gap is particularly relevant to the ED setting, where a rapid synthesis of information, often within seconds to minutes, informs critical decisions [17,18]. As a first step in determining whether LLMs might be suitable for this task, it is essential to systematically compare ED-specific LLM-generated one-liners against physician-authored one-liners.

In this study, we evaluated the ability of an LLM to generate ED-specific, one-liner summaries by synthesizing information from clinical notes and imaging reports spanning prior hospital encounters, using an experimental setup that reflects the real-world cognitive demands of ED physicians during patient encounters. To assess the quality of the LLM-generated summaries, we conducted a blinded evaluation in which emergency physicians compared LLM outputs to physician-authored summaries side by side. Each summary was rated along three core dimensions: accuracy, completeness, and clinical utility.

## Methodology

### Ethics statement

This study was reviewed and approved by the Office for Protection of Human Subjects (OPHS) at the University of California, Berkeley under protocol number 2025-03-18346. The study was granted exemption from full committee review under Federal and UC Berkeley exemption categories 4 and 7, effective May 28, 2025. All research procedures were conducted in accordance with the principles outlined in the Belmont Report and under UC Berkeley’s Federal wide Assurance #00006252. Informed written consent was obtained from all participants prior to their involvement in the study.

### Overview

This study was conducted at the University of California, San Francisco (UCSF), in collaboration with the University of California, Berkeley. Encounters were drawn from the UCSF Information Commons, a repository of deidentified clinical data certified for research use [19]. We followed the STROBE guidelines for observational studies [20]. We additionally followed the TRIPOD-LLM reporting guideline for studies using large language models in healthcare settings [21].

### Cohort selection

We included ED encounters between March 1, 2022, and March 31, 2024 for patients aged ≥18 years with at least one prior UCSF Medical Center inpatient admission. Encounters required (1) a discharge summary from an inpatient admission and (2) an ED provider note from a subsequent visit, ensuring longitudinal clinical context. Encounters were excluded if (1) or (2) were incomplete or if the ED note lacked a clear, identifiable one-liner in the HPI section. We prioritized fully documented encounters to enable evaluation under standardized conditions.

To ensure a breadth of clinical presentations, we used two-tiered stratified sampling: rare chief complaints (occurring in <10 encounters, e.g., rash, hematuria) were fully retained, while common presentations (e.g., chest pain, dyspnea, abdominal pain) were proportionally sampled according to their prevalence distributions. Each encounter was randomly assigned to a unique reviewer to avoid duplicating ratings.

Prior to analysis, a quality control review of all completed evaluations was performed. Evaluations were excluded if the standardized chief complaint used to prompt the LLM was discordant with the chief complaint reflected in the physician-authored one-liner, as this discordance precluded a valid direct comparison. Of 300 sampled encounters, 111 received completed physician reviews, as each reviewer was assigned between 1 and 10 non-overlapping cases. Of these 111 completed evaluations, 12 were excluded due to chief complaint discordance between the standardized LLM input and the physician-authored one-liner, yielding a final analytic sample of 99 unique ED encounters. This flow is depicted in S2 Fig(A).

### LLM-based summary generation

Using UCSF’s secure, HIPAA-compliant Versa API on Microsoft Azure, we prompted the LLM (OpenAI GPT-4o; temperature = 0.1 to balance determinism and lexical variability; other settings default) to generate ED one-liner summaries in two steps. First, using note-title heuristics (S4 Table), we prompted the LLM to select notes most relevant to the ED chief complaint, which was provided from a structured data field containing triage nurse free text. Available note types included discharge summaries, progress notes, consult notes, History & Physical (H&P) notes, imaging reports, imaging reports, echocardiogram reports (cardiac ultrasound), and ECG reports (electrocardiogram tracings). We provided the most recent note of each specified type from the most recent prior inpatient admission preceding the index ED encounter; no fixed time cutoff was applied. The model selected all applicable notes (up to seven, one per note type). The notes selected by the LLM for each encounter were recorded and subsequently analyzed to characterize selection patterns across chief complaint categories. This step reduced computational resources and enabled transparency regarding note selection rationale.

We initially used zero-shot prompting (prompting without examples or demonstrations) but observed substantial temporal confusion, with the model incorrectly attributing prior events to the index encounter. We therefore adopted a few-shot prompting (prompting with a few examples) approach. After grouping cases by chief complaint, we used sentence-transformer representations to embed the discharge summary and chief complaint text. The three most semantically similar cases (k = 3) were retrieved as exemplars, each containing a physician-authored one-liner. These exemplars were inserted into the prompt as contextual examples, allowing the model to generate similarly structured summaries. The full prompt structure is shown in S4 Table.

Pilot testing revealed inconsistent use of standard medical abbreviations (e.g., “SOB” for shortness of breath, “HTN” for hypertension), reducing brevity and potentially signaling LLM authorship. We addressed this by adding a medical abbreviation guidance document [22] to the prompt (S3 Table).

### One-liner summary evaluation

Twenty-one emergency physicians (12 attendings, 9 residents; S1 Table) participated in blinded evaluation of one-liner summaries, each reviewing between 1 and 10 non-overlapping cases. Each participant reviewed cases using a custom web-based platform (Streamlit) simulating a simplified ED chart interface. For each encounter, reviewers received a standardized packet containing patient demographics (age, sex), chief complaint, and the same recent notes provided to the LLM (one of each specified type, up to seven total). They were then shown two anonymized one-liner summaries side by side—one written by the original ED physician and one LLM-generated—presented in randomized order. Both physician-authored and LLM-generated one-liners were constrained to a single sentence, though length varied with patient complexity and breadth of relevant medical history. Physician one-liners were extracted from the opening of the HPI section of the ED provider note using a regular expression pattern and manually verified across all 300 sampled encounters. The LLM was explicitly prompted to generate a single-sentence summary. Reviewers were blinded to authorship. Before rating, all participants completed a brief calibration session with detailed instructions covering the platform and metrics. Reviewers rated each summary using 5-point Likert scales in three domains:

- Accuracy: Is the information factually correct and free of fabrication?
- Completeness: Does it capture all clinically relevant information?
- Clinical utility: How informative is it for decisions in a real-world ED workflow?

Reviewers also indicated a preference between the two summaries (LLM, human, or tie) and could optionally provide a free-text rationale.

### Statistical analysis

To evaluate the LLM’s behavioral rationale, we identified which note types the model incorporated when generating each summary. For each summary, we calculated the number and proportion of available notes utilized, then stratified note selection patterns by chief complaint category grouped into overarching clinical systems. We characterized selected note quantity and word counts using descriptive statistics. We performed Kruskal-Wallis H-tests to assess whether note selection varied significantly across chief complaint categories. Selection rates for individual note types were calculated as the percentage of encounters where each was utilized.

Reviewer preference data were summarized as win rates, defined here as the proportion of encounters in which reviewers favored one summary type over the other. Win rates are reported as percentages alongside statistical comparisons of Likert ratings. For each summary, the three domain scores were averaged to yield an overall quality score (S1 Appendix). Reviewer ratings were analyzed using linear mixed-effects models with summary type (LLM vs. physician) as a fixed effect and reviewer as a random intercept to account for rating clustering. A random intercept for case was initially specified but dropped after contributing negligible variance. The inclusion of reviewer as a random effect was chosen specifically to account for the between-rater differences in rating stringency that are inherent to a single-reviewer-per-case design; this approach partitions rater-level variance before estimating the effect of summary type, providing a more rigorous estimate of the LLM vs. physician difference than a fixed-effects approach would allow [23]. Model estimates were obtained using restricted maximum likelihood, and p-values were computed via Satterthwaite’s degrees-of-freedom approximation [22]. Estimated marginal means and 95% CIs were derived for each summary type within each domain. All tests were two-tailed with α = 0.05. Model residuals were inspected to confirm normality and homoscedasticity assumptions. Free-text justifications were reviewed by N.G. and M.M. to verify that each reviewer’s stated rationale was internally coherent with their summary preference and reflected attributes evident in the selected summary. Clinician-participants completed evaluations remotely via a secure web-based platform on their own time, in a single uninterrupted session. The three evaluation domains, accuracy, completeness, and clinical utility, and the 5-point Likert scale format were adapted from prior LLM clinical summarization studies 11, 13, with descriptors tailored to the ED one-liner context (S5–S7 Tables).

To understand how note characteristics influenced summary quality, we first compared physician and LLM summary performance relative to individual note type lengths using Pearson correlation coefficients. Second, we evaluated how specific note types included in the LLM prompt affected LLM summary quality. For encounters where specific note types were available, we compared LLM summary quality between cases where these notes were included versus excluded, reporting differences using Cohen’s d effect size. Note analyses were performed in Python 3.11; reviewer rating analyses in R version 4.3.1.

### Qualitative analysis

We conducted rapid content analysis of free-text responses to identify key themes [24]. Free-text data were systematically reviewed by NG and MM. Using a descriptive analytic approach, we summarized content within each open-ended item and grouped similar responses into thematic categories through both deductive coding (based on the three evaluation domains) and inductive coding to capture emergent themes. We used a matrix to organize and compare responses across participants and questions. Thematic summaries were refined through iterative team discussion, and representative quotes were identified to illustrate major findings.

Rapid content analysis was conducted by N.G. and M.M. M.M. is a practicing emergency physician, informaticist, and health services researcher. Her clinical expertise informed the interpretation of reviewer preferences but may have foregrounded clinical salience in theme identification. N.G. is a medical student and computer scientist. Her computational background supported systematic analysis, while potentially orienting interpretation toward structured patterns. Both investigators reflected on how their experiences and prior assumptions shaped their interpretation of participant narratives. They used memo-ing, team discussion, and review of discrepant cases to challenge emerging assumptions during analysis.

## Results

### Cohort characteristics

Of 10,631 ED encounters, 3,984 encounters met the inclusion criteria. A random sample of 300 encounters was selected for evaluation. Each physician reviewed 1–10 non-overlapping cases, resulting in 111 completed evaluations. Reviewers assessed both summaries exclusively against the same standardized extracted note packet. To avoid biasing comparisons in favor of the LLM—which generated its summary solely from the standardized documentation—12 evaluations were excluded when the physician-authored one-liner reflected a chief complaint that differed from the standardized LLM input, yielding a final analytic sample of 99 unique ED encounters with 99 unique patients.. Among the notes from the most recent prior inpatient admission and provided to the LLM for selection, nearly all patients contained progress notes (99%), H&P notes (98%), and imaging reports (98%). ECGs were present in 87% of encounters, consult notes in 64%, and echocardiogram reports in 56%. The mean (SD) length of all notes combined per case was 5,598 (2,314) words, with 6.0 (range, 2–7) distinct note types included. Note length ranged from 36 (32) words for ECG reports to 2,310 (1,263) words for discharge summaries, with intermediate lengths for imaging (124 [117] words), echocardiograms (677 [109] words), consult notes (854 [629] words), progress notes (917 [1,010] words), and H&P notes (1,332 [770] words)

### LLM-based summary generation

The LLM requested a mean (SD) of 3.73 (0.67) notes (median: 4.0, IQR: 3.0-4.0), utilizing 53.2% (9.5%) of available notes. H&P notes were nearly universally requested (97.0%), followed by imaging reports (81.8%). Consult notes were included in 44.4% of cases, while cardiac-specific notes (ECG: 21.2%, echocardiogram: 9.1%) and progress notes (19.2%) were selectively utilized. Note selection patterns varied significantly by chief complaint category (Kruskal-Wallis H = 17.29, p < 0.001). Cardiopulmonary presentations required the most comprehensive notes (shortness of breath: 4.9(0.3) notes), with universal inclusion of cardiac studies (ECG: 100%, echocardiogram: 88% for shortness of breath). In contrast, musculoskeletal complaints required fewer notes (back pain: 3.2 (0.5) notes) and appropriately excluded cardiac notes. Psychiatric evaluations demonstrated high utilization of progress notes (75%) and consult notes (100%), while avoiding imaging and cardiac studies entirely (Table 1).

**Table 1:** LLM note selection rates by the top 13 chief complaints.

Category	Chief Complaint	N	Mean		Note Selection Rate (%)					
				DS	HP	PN	ECG	IM	EC	CN
Cardiopulm.	SOB	8	4.9	100	100	0	100	100	88	0
	Chest Pain	4	4.0	100	75	0	100	100	25	0
GI	Abd. Pain	23	3.6	100	96	0	0	100	0	65
General	Fever	3	3.7	100	100	67	0	67	0	33
	Referral	3	3.0	100	100	67	0	0	0	33
	Fall	2	3.5	100	100	50	0	100	0	0
MSK	Back Pain Foot	4	3.2	100	100	0	0	100	0	25
	Pain	2	3.0	100	100	0	0	100	0	0
Neuro	Headache	3	3.7	100	100	0	0	100	0	67
	Seizures	3	4.7	100	100	0	100	100	0	67
	Dizziness	3	4.0	100	100	0	100	100	0	0
	Syncope	2	4.0	100	100	0	50	100	50	0
Psych	Psych Eval	4	3.8	100	100	75	0	0	0	100

DS = Discharge Summary; HP = History and Physical; PN = Progress Notes; ECG = Electrocardiogram; IM = Imaging report; EC = Echocardiogram; CN = Consult Note. SOB = Shortness of Breath; Abd. = Abdominal; Cardiopulm. = Cardiopulmonary; Neuro = Neurological; MSK = Musculoskeletal; Psych = Psychiatric.

Mean represents the average number of notes selected per encounter. N = number of encounters for the given chief complaint.

### One-liner summary evaluation

Across all 99 cases, LLM-generated one-liners were preferred over physician-authored one-liners in 50 cases (50.5%); physician-authored one-liners were preferred in 38 cases (38.4%); 11 cases (11.1%) were rated equally. Excluding ties, the LLM win rate was 56.8% (95% CI, 46.4–66.7%).

**Fig 1:**
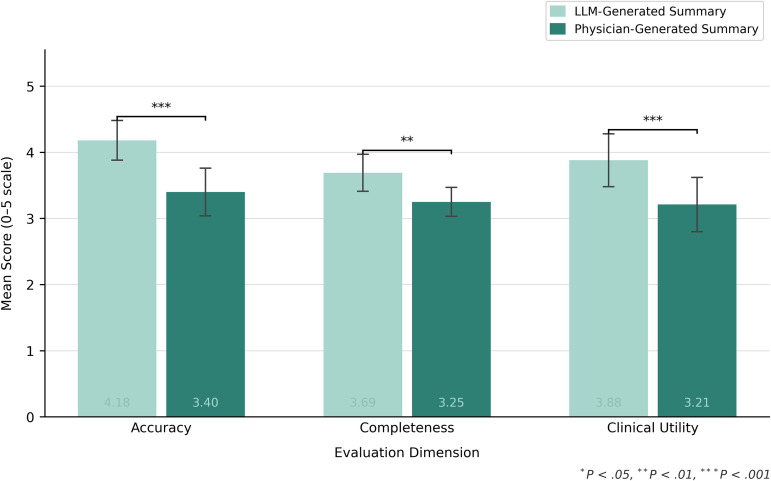
Comparison of LLM-generated and physician-authored one-liner summaries across evaluation dimensions. Mean scores (± 95% CI) for LLM-generated summaries (light teal bars) and physician-authored summaries (dark teal bars) on a 5-point Likert scale across accuracy, completeness, and clinical utility. Significance was based on the model-estimated confidence interval of the paired mean difference.

In mixed-effects models adjusting for reviewer as a random effect, LLM-generated summaries scored 0.78 points higher for accuracy (95% CI 0.497–1.059; p = 1.98 × 10 ⁻ ⁷), 0.43 points higher for completeness (95% CI 0.152–0.716; p = 0.0030), and 0.67 points higher for clinical utility (95% CI 0.360–0.973; p = 3.28 × 10 ⁻ ⁵). The overall composite score similarly favored the LLM (β = 0.626, SE = 0.131; 95% CI 0.369–0.884; p = 3.82 × 10 ⁻ ⁶). Model-estimated marginal means (± 95% CI) were 4.18 (3.83–4.54) vs 3.40 (3.05–3.76) for accuracy, 3.69 (3.37–4.00) vs 3.25 (2.94–3.57) for completeness, and 3.88 (3.47–4.28) vs 3.21 (2.81–3.61) for clinical utility, for LLM-generated and physician-authored summaries respectively (Fig 1). Case-level variance was estimated at zero (reflecting the single-reviewer-per-case design), while reviewer-level variance was non-zero (SD ≈ 0.65–0.80), indicating individual differences in rating stringency. Overall, LLM-generated summaries were judged by physician reviewers as more accurate, complete, and clinically useful.

### Source note characteristics and summary performance

#### Impact of note length between physician vs LLM.

For LLM-generated summaries, overall note length was not associated with accuracy (r = 0.053, p > 0.05), completeness (r = 0.039, p > 0.05), or clinical utility (r = 0.094, p > 0.05). In contrast, physician-authored summary completeness increased with longer notes (r = 0.229, p = 0.023). Stratifying our analysis by specific note type revealed that for LLM-generated summaries, longer H&P notes were associated with higher accuracy (r = 0.243, p = 0.016), whereas longer progress notes were negatively correlated with completeness (r = 0.229, p = 0.023) and overall quality (r = 0.210, p = 0.038).

#### Impact of selective note inclusion on LLM-generated summaries.

The effect of selective note inclusion by the LLM was evaluated. When imaging reports were both available and selected by the LLM, summary quality scores were higher (mean 4.05, SD 0.93) compared with encounters where imaging reports were available but not included (mean 3.58, SD 1.14; Cohen’s d = 0.49; p = 0.078).

**Fig 2:**
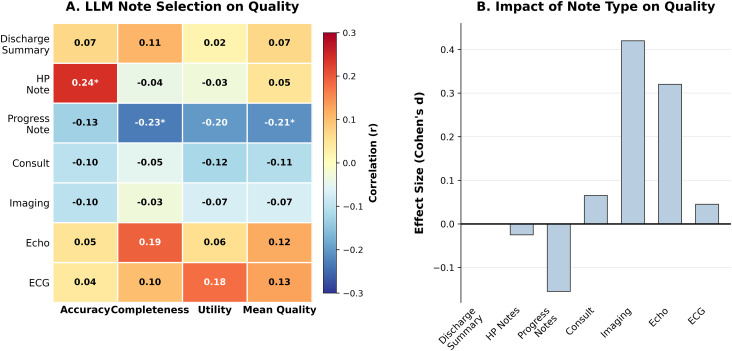
Impact of note characteristics on LLM summary quality. **(A)** Correlations (*r*) between individual note type lengths (decreasing in length from top to bottom of the matrix) and quality scores across domains (accuracy, completeness, clinical utility, and mean quality). **(B)** Effect sizes (Cohen’s *d*) showing the impact of explicitly including specific note types on LLM-generated summary quality compared to when those note types were available but not included. Asterisks indicate statistically significant correlations (p < 0.05).

Fig 2A shows correlations between individual note type lengths and LLM-generated summary quality across domains. Fig 2B displays the effect of explicitly including individual note types in the LLM input. Imaging reports (Cohen’s d = 0.42) and echocardiograms (Cohen’s d = 0.32) were associated with improved summary quality compared to encounters where these notes were available but not included, while inclusion of progress notes had a small negative effect.

### Qualitative analysis

Fifty-five paired evaluations had free-text commentary in which reviewers explained their preferences between LLM- and physician-authored summaries. The major themes emerging from these explanations are shown in Table 2.

**Table 2:** Themes from reviewer free-text explanations of one-liner preferences.

Theme	Representative Quotes
Importance of factual reliability as measured by alignment with source documentation	“Does not have invented PMH; accuracy matters even if incomplete.” *(preferred LLM)* “She’s not actually on oxygen which is why I don’t like A.” *(preferred LLM)* “Both of them indicate he had transverse sinus thrombosis, which it didn’t actually appear to have on final MRI (though there was initial concern for it).” *(equal preference)*
Inclusion of clinical context relevant to presenting complaint can help guide differential diagnosis	“History of MDR bacteriuria relevant to abdominal pain.” *(preferred Physician)* “Colors our approach to this person’s abdominal pain.” *(preferred Physician)* “Mentions chest pain with abdominal pain, changing DDX.” *(preferred Physician)* “Misses critically important information about the patient’s history with arrhythmia, which could lead to a dizziness that may have preceded the fall.” *(preferred LLM)* “Includes residual stroke deficits relevant to the presentation.” *(preferred LLM)*
Accurate references to recency and timing of key clinical events are essential	“Gives the specifics of the procedure in a very concise way and actually gets the date right.” *(preferred LLM)* “Many of the problems in the one-liner were from a recent hospitalization (as opposed to not knowing how recent they were).” *(preferred LLM)*
Added value of actionable information supporting triage or management decisions	“Would prompt me to see the patient sooner since it mentioned unstable vitals.” (*preferred Physician)* “Includes who to involve as consultants.” *(preferred Physician)* “Includes pertinent information about the specific form of cancer, location of mets, and a recent admission for sepsis which significantly changes my initial approach to this pt” *(preferred LLM)*

During the content analysis, four major themes emerged: (1) Importance of factual reliability as measured by alignment with source documentation, (2) Inclusion of relevant clinical context to presenting complaint can help guide differential diagnosis, (3) Accurate references to recency and timing of key clinical events are essential, and (4) Added value of actionable information supporting triage or management decisions (Table 2). Reviewers often praised LLM-generated summaries for their factual reliability and adherence to charted information. Physician-authored summaries were favored when they had richer contextual framing, including key comorbidities and diagnostic nuances (“History of MDR bacteriuria, relevant to abdominal pain”) that prompted actionable items. Reviewers especially valued summaries that guided clinical prioritization or next steps, for example, noting key contextual details (e.g., variceal banding) that informed which consultants to involve early. In some cases, reviewers found the LLM and physician summaries equally useful but non-overlapping, highlighting complementary strengths and suggesting that combining them could yield more clinically salient, useful summaries: “Both statements contain something that the other is missing which would be helpful (ex: B leaves out the fact patient is not taking AC [anticoagulation] for known DVT [deep venous thrombosis], A leaves out what specific substance, etc.)”.

## Discussion

In this study, we found that, with domain-specific context, LLM-generated one-liner summaries received higher ratings than physician-authored summaries across accuracy, completeness, and clinical utility in a blinded retrospective comparison. Secondary analysis showed that within LLM-generated summaries, selective inclusion of structured or diagnostically relevant notes, particularly imaging reports and echocardiograms, was associated with higher summary quality compared to encounters where those note types were available but not selected. An equivalent analysis could not be performed for physician-authored summaries, as the notes accessed by treating physicians during the original clinical encounter were not tracked. While reviewers felt physician summaries offered contextual nuance, LLM summaries were more factually aligned with the chart, temporally accurate, and inclusive of key details.

While prospective validation is required before any clinical conclusions can be drawn, our findings suggest several directions worth exploring. The complementary error profiles between LLM- and physician-authored summaries suggest potential for synergy. The LLM’s performance remained stable regardless of note volume, suggesting robustness to information density and fragmentation—conditions contributing to cognitive overload and fatigue in ED physicians [25,26]. This stability under high-volume input is especially advantageous in the ED, which requires longitudinal synthesis of clinical information. A hybrid workflow would leverage both strengths: the LLM would ensure completeness and neutral phrasing, while the physician would contextualize information relevant to the clinical presentation [27]. However, any such deployment must account for the risk of automation bias — the tendency for clinicians to over-rely on algorithmically generated outputs, potentially accepting LLM summaries without adequate critical review [28]. In the ED setting, where time pressure is high and cognitive load is substantial, this risk may be particularly pronounced. Safeguards such as clinician review of temporal claims, explicit flagging of LLM-generated content, and ongoing monitoring of summary accuracy will be essential to mitigate this risk in any prospective deployment. In the proposed hybrid model, the LLM would not produce a summary alongside a separate physician-authored one for reconciliation; rather, it would generate a validated summary with mechanisms for source attribution — such as excerpt-level citations — that allow the physician to efficiently verify key clinical claims. In this way, the model is designed to reduce net cognitive load by front-loading information synthesis, not to add a second document for the clinician to process.. In this way, LLM-generated ED one-liners could hypothetically reduce chart review time, support rapid cognitive framing during high-acuity encounters, enhance handoff completeness, and ease documentation burden — though these potential benefits remain untested and require prospective evaluation in live clinical settings before any conclusions about real-world impact can be drawn. By front-loading information synthesis, LLMs might enable clinicians to focus on higher-order reasoning and patient interaction, potentially lessening burnout, which remains pervasive in emergency medicine [7,25,29–32]. While our study demonstrates that LLMs can integrate clinical information across encounters, their impact on clinical care requires prospective evaluation.

Prior work using LLMs to produce long-form clinical summarization [11–13,33]. has largely focused on single-encounter summarization tasks such as discharge summaries [14]. Our findings extend and contextualize prior work on LLM-based clinical summarization. Van Veen et al. evaluated long-form summarization of radiology reports, progress notes, and doctor-patient dialogue, and found that adapted LLMs were rated equivalent or superior to medical expert summaries in 81% of cases, a directionally consistent finding to ours, though their task involved single-encounter, long-form summarization rather than the cross-encounter, single-sentence synthesis required for ED one-liners. Williams et al., in the most methodologically comparable study, found LLM- and physician-generated discharge summary narratives to be of comparable overall quality in a blinded evaluation at UCSF. By contrast, our study found a more pronounced LLM advantage across all three domains, particularly for accuracy (4.18 vs 3.40, β = 0.78), a difference we attribute to the use of kNN few-shot prompting and selective multi-encounter note inclusion, which substantially reduced temporal grounding errors compared to the zero-shot approaches used in prior work. Importantly, while Williams et al. focused on discharge summaries generated from single ED encounters, our task required synthesis across multiple prior inpatient admissions, representing a more cognitively demanding and clinically novel summarization challenge. Finally, o. Importantly, we allowed the model to select its own source documents, mirroring clinical information triage and avoiding degradation from indiscriminate input ingestion. We thus extend the literature by showing how prompting strategy and selective note inclusion impacted summary quality.

The LLM’s performance critically depended on how the input was structured, and temporal grounding errors represent a clinically important safety concern that warrants explicit attention. In zero-shot settings, we observed two recurring error types: (1) timeline collapse, in which chronic conditions were misrepresented as acute complaints, and (2) result misattribution, in which prior test results were incorrectly attributed to the index encounter. Both error types could plausibly lead to diagnostic error or inappropriate management if deployed without oversight — for example, a chronic finding reframed as new could prompt unnecessary workup, while a prior result misattributed to the current visit could falsely reassure a clinician. Few-shot prompting substantially reduced these errors but did not eliminate them entirely. In any prospective deployment, safeguards should include explicit timestamp anchoring and note hyper-linkage in the prompt, clinician review of temporal claims before acting on summarized content, and systematic logging of cases flagged for timeline inconsistencies to enable ongoing safety monitoring [33]. The kNN few-shot prompting technique improved temporal coherence and clinical specificity, consistent with prior work on domain-specific prompting and retrieval-augmented generation in clinical NLP [31,34]. This highlights that high-quality ED summarization across multiple encounters requires structured context, curated exemplars, and an explicit consideration of time.

Our findings also have implications for note curation. The model demonstrated context-sensitive document selection, incorporating only ~53% of notes while maintaining clinical appropriateness. This selective behavior mirrors expert physician heuristics [35–37] and varied with chief complaint; for example, cardiac documents were nearly always selected for cardiopulmonary presentations and rarely for musculoskeletal or psychiatric cases. This “clinical mimicry” is critical for accuracy, efficiency, and broader health system implementation, where token costs accumulate rapidly; real-world deployment will require selective note strategies to remain operationally viable [38]. We found that high-yield notes (H&P notes, imaging reports) disproportionately improved summary quality, while verbose progress notes reduced it. These effects may reflect length-related signal dilution and recency or “primary source” bias, where more recent or central documents disproportionately influence summarization [39,40]. These findings highlight that targeted retrieval of concise, clinically salient documents—rather than indiscriminate text ingestion—is essential for cost-effective, high-quality summaries.

Despite context-sensitive note selection, contemporary LLMs (e.g., GPT-4o) offer little visibility into how they prioritize clinical documents [41], which would obscure transparency for ED physicians attempting to quickly verify information sources and accuracy. The reduced cognitive load and improved efficiency of automated one-liner generation can only be realized if ED physicians can rapidly validate model-derived content, as they lack time to cross-check autogenerated summaries against the full chart. Our analyses suggest a practical solution: enable the model to surface excerpt-level citations underlying each piece of information. Such source transparency would allow clinicians to quickly verify key details without navigating long documents, balancing efficiency with oversight. As health systems consider deploying LLM-assisted chart summarization [42], incorporating transparent attribution mechanisms will be essential to establish clinician trust and enable safe adoption in time-pressured environments.

## Limitations

First since each case was evaluated by a single reviewer, interrater reliability could not be formally estimated. The reviewer-level random effect (SD ≈ 0.65–0.80) confirmed substantial individual differences in rating stringency, indicating that some of the observed variance in ratings reflects rater-specific tendencies rather than true differences in summary quality. This is particularly consequential for the completeness domain, where the effect size was smallest (β = 0.43; 95% CI 0.14–0.74) and therefore most vulnerable to rater noise. While predefined Likert scale anchors and free-text justification analysis were used to support scoring consistency, these measures cannot substitute for formal reliability estimation. Readers should interpret the effect sizes — especially for completeness — with this uncertainty in mind. Second, one of the primary limitations of this study was the structural asymmetry inherent to the evaluation design. Physician-authored one-liners were composed during live clinical encounters, where treating physicians had access to bedside observation, verbal patient history, and real-time clinical judgment — none of which was captured in the extracted note packets provided to reviewers. The LLM, by contrast, generated its summaries exclusively from those extracted notes, which also served as the reviewer’s reference standard. This creates a disadvantage for physician-authored summaries: information obtained at the bedside but absent from the notes would appear unsupported to reviewers, while the LLM’s note-derived content would appear well-grounded. To mitigate this bias, we performed a pre-analysis quality control step in which 12 of 111 evaluations (10.8%) were excluded due to chief complaint discordance — the primary mechanism by which this asymmetry manifested in our dataset. The remaining 99 cases represent encounters where both summaries were anchored to equivalent input context. Nevertheless, residual information asymmetry cannot be fully eliminated in a retrospective design, and effect sizes reported here should be interpreted as likely upper bounds on the true advantage of LLM-generated summaries under conditions of equivalent information access. Future evaluations should provide reviewers with the full clinical context available to the treating physician, or restrict the comparison to information present in the extracted notes. Third, Second, generalizability is limited by our single-center design and inclusion criteria. Requiring a prior inpatient admission and complete documentation likely selected for complex, documentation-rich encounters. While these findings may be less generalizable to community EDs, pediatric populations, or leaner EHR ecosystems, it is precisely these clinically complex cases, with extensive longitudinal records spanning multiple encounters, for which automated summarization offers the greatest potential value. Prospective evaluation across diverse clinical settings is needed to establish broader applicability. Additionally, our stratified sampling design, while ensuring breadth of clinical presentations, resulted in small subgroups for rare chief complaints, precluding a reliable breakdown of summary quality by presentation type. Future studies with larger samples should examine whether LLM performance varies systematically across chief complaint categories. Finally, while we did not test alternative prompting or fine-tuning strategies, these would likely match or improve the performance achieved with this minimally optimized, out-of-the-box model.

## Conclusions

In this blinded evaluation, LLM-generated one-liner summaries achieved higher scores for accuracy, completeness, and clinical utility than physician-authored summaries. These findings suggest that, with structured prompting and clinical oversight, generative models can produce one-liner summaries that are rated as more accurate, complete, and clinically useful than physician-authored summaries in a controlled retrospective comparison. Prospective validation in live ED workflows is a necessary next step before these tools can be considered for clinical deployment [10,11]. Implementation must prioritize transparency and safety, allowing these tools to augment, not replace, clinical reasoning [9].

## Supporting information

S1 Appendix Summary of the kNN few-shot summarization pipeline with dynamic note selection, including stepwise procedure and operational safeguards. (DOCX)

S2 Appendix Full instructions provided to physician participants prior to initiating the blinded evaluation, as displayed in the Streamlit-based interface. (DOCX)

S1 Fig Instructional image displayed to participants in the evaluation interface, shown with redacted patient-sensitive information. (DOCX)

S2 Fig Study methodology and LLM pipeline: (A) systematic cohort selection from electronic health records using inclusion criteria and stratified sampling, and (B) LLM-based pipeline for generating one-liner clinical summaries from multisource clinical data using a k-nearest neighbors approach. (DOCX)

S1 Table Demographic and professional characteristics of the 21 emergency physician reviewers participating in the blinded evaluation. (DOCX)

S2 Table Patient encounter characteristics for the final analytic sample of 99 ED encounters. (DOCX)

S3 Table Retrieval and generation hyperparameters used in the LLM-based summarization pipeline. (DOCX)

S4 Table Prompt templates used for the note selection and one-liner generation steps of the LLM pipeline. (DOCX)

S5 Table Accuracy evaluation criteria and Likert scale descriptors provided to physician reviewers. (DOCX)

S6 Table Completeness evaluation criteria and Likert scale descriptors provided to physician reviewers. (DOCX)

S7 Table Clinical utility evaluation criteria and Likert scale descriptors provided to physician reviewers. (DOCX)
