# Supplementary material for: Large language model-generated clinical summaries in emergency departments: A blinded comparison study
Source: PLOS Digit Health. 2026 Jul 9;5(7):e0001491. doi: 10.1371/journal.pdig.0001491 (PMC13349196; doi:10.1371/journal.pdig.0001491)
Supplement: S1 Appendix — (DOCX) [file pdig.0001491.s001.docx]

# **S1 Appendix. kNN Few-Shot Summarization With Dynamic Note Selection (Method Summary)**

**Overview** We implement a two–stage pipeline that (1) retrieves *k* clinically similar, high– quality one–liner examples via dual–source embeddings and (2) prompts an LLM to *first* select which note types to review (dynamic input curation) and *then* generate a temporally coherent ED one–liner with abbreviation guidance and few–shot exemplars.

## **Stepwise Procedure**

1. **Candidate pool:** Start with ED encounters that contain (a) a chief complaint (CC), (b) prior EHR notes (e.g., discharge summary, progress, H&P, imaging, ECG, echo, consult), and (c) a human one–liner (for exemplars).
2. **Embeddings:** Compute Bio ClinicalBERT embeddings for (i) the current case CC and (ii) the current discharge summary (if present). Do the same for all candidate exemplars.
3. **Similarity:** Compute cosine similarities for CC and discharge summary separately; combine with equal weights (0.5/0.5) to yield a single similarity score per candidate.
4. **kNN selection:** Select the top *k* = 3 most similar cases with ground–truth one–liners to use as few–shot exemplars (fall back to fewer if needed).
5. **Dynamic note selection:** Present the LLM with the list of *available* note types for the current case and ask it to return a *comma–separated list* of the notes it wants to read, guided by the chief complaint (hard rule: always include discharge summary if available). Filter out unavailable notes.
6. **Input assembly:** For each requested note, extract content (with date), and concatenate into the *PAST Medical Records* section.
7. **Few–shot context:** Append the *k* exemplar pairs (Example Chief Complaint, Expected Summary) to the prompt.
8. **Abbreviation guidance:** Provide a compact list mapping terms to standard ED abbreviations to enforce concise, specialty–consistent phrasing.
9. **Generation:** Prompt the model with a strict temporal framing: summarize *past* history and *end* with the *current* chief complaint (unaltered), avoiding inference about present illness.
10. **Operational safeguards:** Batch processing (default size 10), GPU use for embeddings, retry logic for API calls (up to 3), caching of embeddings, and default fallbacks if the selection step fails (e.g., at least Discharge Summary).

**Rationale** Dual–source embeddings (CC + Discharge Summary) improve case retrieval relevance; dynamic note selection constrains input volume and focuses on clinically high– yield sources, reducing token cost and improving scalability for health–system deployment. Abbreviation guidance enforces ED style, while few–shot exemplars improve temporal coherence and structure.
