# Supplementary material for: Large language model-generated clinical summaries in emergency departments: A blinded comparison study
Source: PLOS Digit Health. 2026 Jul 9;5(7):e0001491. doi: 10.1371/journal.pdig.0001491 (PMC13349196; doi:10.1371/journal.pdig.0001491)
Supplement: S2 Appendix — (DOCX) [file pdig.0001491.s002.docx]

# **Appendix S2. Instructions Provided to Physician Participants**

Prior to initiating the evaluation, participants were shown the following on-screen instructions in the Streamlit-based application used for the study.

# **Study Overview**

You will be reviewing patient cases from the emergency department. For each case, you’ll complete a summarization evaluation task.

# **Patient Record Review**

For each case, you will first review the patient’s medical record, which includes:

- Current chief complaint
- Relevant clinical documentation (e.g., ECG, echocardiogram) typically available during ED workflow

You may review the patient’s record by clicking on the appropriate tabs at the top of the screen or by using the sidebar while completing the evaluation task. Please take your time to review the materials thoroughly before proceeding.

# **Summarization Task**

After reviewing the patient record, you will evaluate two different one-liner summaries of the case. These are intended to represent the type of one-liner you would write as the ED provider during the patient’s current visit.

You will rate each summary on three dimensions: Accuracy, Completeness, and Clinical Utility (S5-7 Tables).

**Accuracy**: Is the information in the summary factually correct (i.e., free from false or fabricated content)?

**Completeness**: Does the summary capture all critical and relevant historical or contextual information?

**Clinical Utility**: Would this summary be helpful in a real-world clinical workflow?
