# Supplementary material for: Large language model-generated clinical summaries in emergency departments: A blinded comparison study
Source: PLOS Digit Health. 2026 Jul 9;5(7):e0001491. doi: 10.1371/journal.pdig.0001491 (PMC13349196; doi:10.1371/journal.pdig.0001491)
Supplement: S1 Fig — (DOCX) [file pdig.0001491.s003.docx]

# **Navigation and Progress**

You may navigate between patient encounters using the buttons at the bottom of each page. Your progress is saved automatically upon submission of each evaluation.

*Note: An instructional image (see below) was also displayed in the interface. Participants could not proceed without viewing this screen.*


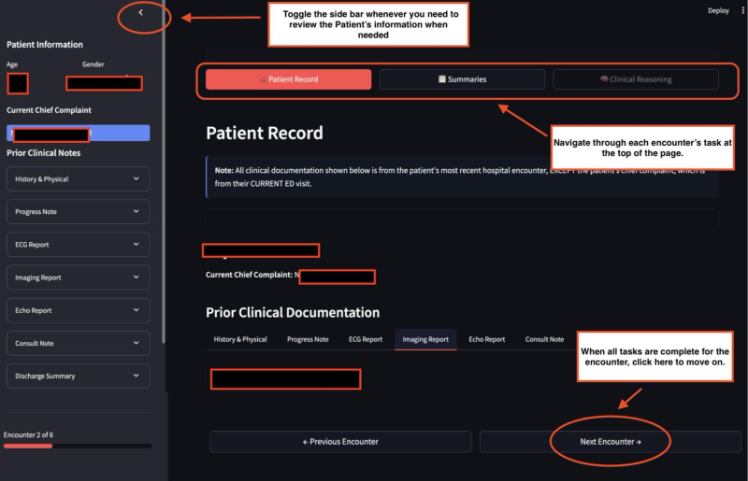


**S1 Fig: Instructional Image with Redacted Patient-Sensitive Information**
