# Supplementary material for: Large language model-generated clinical summaries in emergency departments: A blinded comparison study
Source: PLOS Digit Health. 2026 Jul 9;5(7):e0001491. doi: 10.1371/journal.pdig.0001491 (PMC13349196; doi:10.1371/journal.pdig.0001491)

# **S2 Fig. Study Methodology and LLM Pipeline:** (A) systematic cohort selection from electronic health records using inclusion criteria and stratified sampling, and (B) LLM-based pipeline for generating one-liner clinical summaries from multisource clinical data, with K-nearest neighbors approach.


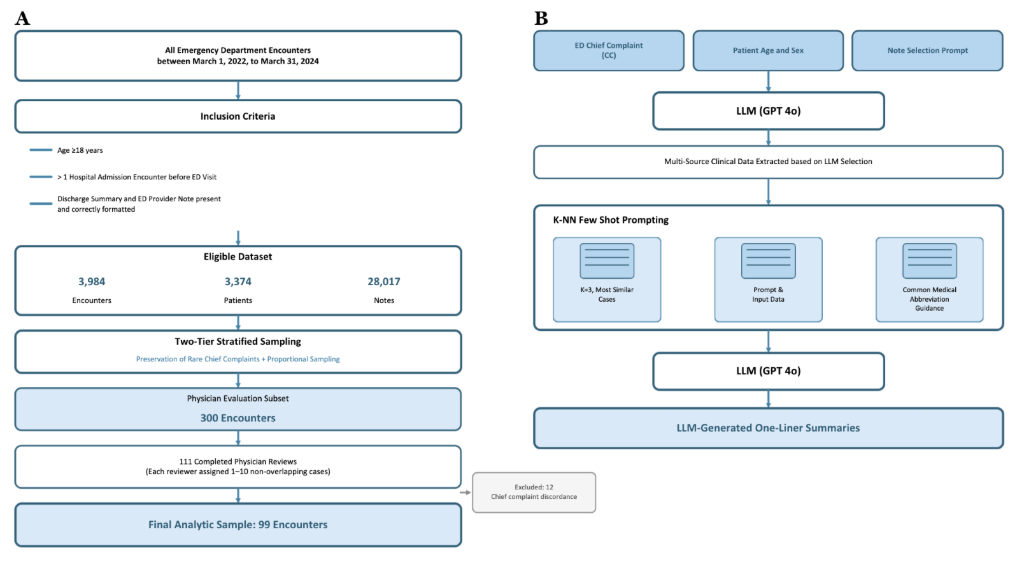

Supplement: S2 Fig — (DOCX) [file pdig.0001491.s004.docx]
