# Supplementary material for: Large language model-generated clinical summaries in emergency departments: A blinded comparison study
Source: PLOS Digit Health. 2026 Jul 9;5(7):e0001491. doi: 10.1371/journal.pdig.0001491 (PMC13349196; doi:10.1371/journal.pdig.0001491)
Supplement: S1 Table — (DOCX) [file pdig.0001491.s005.docx]

| **Characteristic** | **Value** |
| --- | --- |
| **Basic Demographics**  Age, mean (SD), y | 36.3 (9) |
| Age, median (IQR), y | 32.0 (30.0–43.0) |
| Age range, y | 27–55 |
| Sex, No. (%)  Male | 11 (52.3) |
| Female | 8 (38.1) |
| Non-binary | 1 (4.8) |
| Prefer not to say | 1 (4.8) |
| **Professional Characteristics**  Credential type, No. (%)  Attending | 12 (57.14) |
| Resident | 9 (42.84) |
| ER experience, mean (SD), y | 6.8 (6.0) |
| ER experience range, y | 2–23 |
| **AI Experience and Attitudes**  Clinical AI experience level, No. (%)  Minimal | 9 (42.3) |
| Extensive | 4 (19.0) |
| None | 6 (28.6) |
| Moderate | 2 (9.5) |
| Clinical AI openness, No. (%)  Very open | 11 (52.4) |
| Somewhat open | 5 (23.8) |
| Neutral | 4 (19.0) |
| Somewhat resistant | 1 (4.8) |
| AI tools used (Select all that applies), No. (%)  OpenEvidence | 4 (15.4) |
| Perplexity | 2 (7.7) |
| ChatGPT | 2 (7.7) |
| Other AI tools | 1 (3.8) |
| Epic-integrated AI (e.g., Dragon) | 1 (3.8) |
| Claude | 1 (3.8) |
| Gemini/Bard | 1 (3.8) |
