# Supplementary material for: Large language model-generated clinical summaries in emergency departments: A blinded comparison study
Source: PLOS Digit Health. 2026 Jul 9;5(7):e0001491. doi: 10.1371/journal.pdig.0001491 (PMC13349196; doi:10.1371/journal.pdig.0001491)
Supplement: S2 Table — (DOCX) [file pdig.0001491.s006.docx]

| **Characteristic** | **Value** |
| --- | --- |
| Age, mean (SD), y | 57.0 (21.0) |
| Age, median (IQR), y | 62.0 (37.0–74.5) |
| Age range, y  Age group, No. (%) | 18–88 |
| 65+ | 45(45.5) |
| 45–64 | 23(23.2) |
| 30–44 | 19(19.2) |
| 19–29 | 8(8.1) |
| =18  Sex, No. (%) | 4(4.0) |
| Female | 55(55.6) |
| Male  Race/ethnicity (top 5), No. (%) | 44(44.4) |
| White | 39(39.4) |
| Asian | 26(26.3) |
| Other | 15(15.2) |
| Black or African American | 14(14.1) |
| Native Hawaiian or Other Pacific Islander | 2(2.0) |
| Other race (2 categories)  **Clinical Characteristics**  Top 10 chief complaints, No. (%) | 3(3.0) |
| Abdominal pain | 23(23.2) |
| Shortness of breath | 8(8.1) |
| Psychiatric evaluation | 4(4.0) |
| Back pain | 4(4.0) |
| Chest pain | 4(4.0) |
| Referral | 3(3.0) |
| Headache | 3(3.0) |
| Seizures | 3(3.0) |
| Fever | 3(3.0) |
| Dizziness | 3(3.0) |
| Total unique chief complaints  Top 10 ED diagnoses, No. (%) | 48 |
| Chest pain, unspecified type  Acute Abdominal Pain  Acute nonintractable headache, unspecified  Seizure  Abdominal Pain, epigastric  Sepsis, unspecified organism  Abdominal Pain, left upper quadrant  Fever, unspecified cause  Suiciudal ideation  Pneumonia, unspecified organism  Total Unique ED Diagnoses | (5(5.1)  4(4.0)  4(4.0)  3(3.0)  2(2.0)  2(2.0)  2(2.0)  2(2.0)  2(2.0)  2(2.0)  80 |
