# Supplementary material for: Large language model-generated clinical summaries in emergency departments: A blinded comparison study
Source: PLOS Digit Health. 2026 Jul 9;5(7):e0001491. doi: 10.1371/journal.pdig.0001491 (PMC13349196; doi:10.1371/journal.pdig.0001491)
Supplement: S3 Table — (DOCX) [file pdig.0001491.s007.docx]

| **Component** | **Setting / Value** |
| --- | --- |
| Embedding model | Bio ClinicalBERT (CLS embedding) |
| Similarity metric | Cosine similarity |
| Similarity fusion | CC (0.5) + Discharge Summary (0.5) |
| *k* (nearest neighbors) | 3 (fallback to available) |
| Batch size | 10 |
| LLM temperature | 0.1 |
| LLM max tokens (generation) | 4096 |
| Selection retries / Gen retries | up to 3 / up to 3 |
| Abbreviation guidance | Included (domain list) |
| Few–shot exemplars | Included (CC + human one–liner) |
| Defaults on selection failure | Discharge Summary |

| **Prompt** | **Role** | **Content (verbatim)** |
| --- | --- | --- |
| **Note Selection** | System | You are an experienced emergency department (ED) who is preparing to write your note for a patient ED visit. Your task is to decide which medical notes you want to read to create the most accurate and comprehensive summary for your one-liner in your note. You should always start with the discharge summary. |
