# Supplementary material for: Large language model-generated clinical summaries in emergency departments: A blinded comparison study
Source: PLOS Digit Health. 2026 Jul 9;5(7):e0001491. doi: 10.1371/journal.pdig.0001491 (PMC13349196; doi:10.1371/journal.pdig.0001491)
Supplement: S4 Table — (DOCX) [file pdig.0001491.s008.docx]

Prompt Templates

| **Note Selection** | User | Patient basic info: {age}yo {sex} with chief complaint: {chief 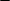complaint} Available notes (respond ONLY with the names of notes you want to see, separated by commas): - Discharge  Summary: {Available/Not available} Progress Notes: {Available/Not available}  - H&P: {Available/Not available} -  Echo: {Available/Not available} Imaging: {Available/Not available} Consult: {Available/Not available} - ECG:  {Available/Not available}  Based on the chief complaint, list  ONLY the note types you need to review (comma-separated, no explanation): |
| --- | --- | --- |
| **One–Liner**  **Generation** | System | You are an experienced emergency department  (ED) physician creating a one-liner for a  NEW patient who has just arrived at the ED. The patient’s past medical records are available to you. Your task is to summarize the patient’s relevant PAST medical history and end with their CURRENT chief complaint that is given with no adjectives about the chief complaint as you can NOT assume anything about their current condition. All notes and medical records provided are from PAST encounters, not the current visit. |


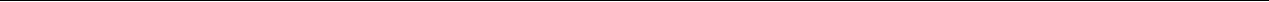


| **One–Liner**  **Generation** | User | Create a concise one-liner summary for a patient who has just arrived at the Emergency Department on {arrival 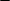date}. The one-liner must:  1. Start with demographic information (age, sex) 2. Include a concise summary of relevant PAST medical history from previous visits/notes 3. End with just CURRENT presenting chief complaint that is not capitilized in the summary and does have additional information regarding the chief complaint: ’{chief complaint}’  IMPORTANT: Everything in the notes is from PAST encounters. The patient is NOW presenting with a NEW complaint:  ’{chief complaint}’.  Use medical abbreviations where appropiate: |
| --- | --- | --- |

{abbreviationguidance}


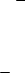
Here are some example summaries for similar cases: {fewshot examples}

Now, create a similar summary for this new case: Current Chief Complaint: {chief complaint} Age: {age} Sex: {sex} Current ED Arrival Date: {arrival date}

PAST Medical Records: {notes content}
