# Supplementary material for: Large language model-generated clinical summaries in emergency departments: A blinded comparison study
Source: PLOS Digit Health. 2026 Jul 9;5(7):e0001491. doi: 10.1371/journal.pdig.0001491 (PMC13349196; doi:10.1371/journal.pdig.0001491)
Supplement: S5 Table — (DOCX) [file pdig.0001491.s009.docx]

**S5 Table: Accuracy Evaluation Criteria**

| **Score** | **Description** |
| --- | --- |
| **5** | Completely accurate; no errors or hallucinated information. |
| **4** | Mostly accurate; minor errors unlikely to impact care. |
| **3** | Partially accurate; includes some fabricated content that may affect care. |
| **2** | Mostly inaccurate; significant falsehoods impacting care. |
| **1** | Dangerous inaccuracies; could result in patient harm. |
