# Supplementary material for: Large language model-generated clinical summaries in emergency departments: A blinded comparison study
Source: PLOS Digit Health. 2026 Jul 9;5(7):e0001491. doi: 10.1371/journal.pdig.0001491 (PMC13349196; doi:10.1371/journal.pdig.0001491)
Supplement: S6 Table — (DOCX) [file pdig.0001491.s010.docx]

**Table S6: Completeness Evaluation Criteria**

| **Score** | **Description** |
| --- | --- |
| **5** | All critical information required for clinical decision-making is included. |
| **4** | Most important elements included; minor omissions present. |
| **3** | Basic information present; several key elements are missing. |
| **2** | Major omissions of important clinical data. |
| **1** | Critically incomplete; unsuitable for clinical use. |
