# Supplementary material for: Large language model-generated clinical summaries in emergency departments: A blinded comparison study
Source: PLOS Digit Health. 2026 Jul 9;5(7):e0001491. doi: 10.1371/journal.pdig.0001491 (PMC13349196; doi:10.1371/journal.pdig.0001491)
Supplement: S7 Table — (DOCX) [file pdig.0001491.s011.docx]

**S7 Table: Clinical Utility Evaluation Criteria**

| **Score** | **Description** |
| --- | --- |
| **5** | Would significantly improve clinical workflow and save time. |
| **4** | Would somewhat improve workflow. |
| **3** | Neutral impact on workflow. |
| **2** | Would somewhat impede workflow. |
| **1** | Would significantly impede workflow or increase workload. |
